# Supplementary material for: β-Hydroxybutyrate elicits divergent metabolic responses between MCF-7 and T47D ER+ breast cancer cells under glucose restriction
Source: bioRxiv. 2026 May 18:2026.05.14.725288. Preprint. [Version 1] doi: 10.64898/2026.05.14.725288 (PMC13228522; doi:10.64898/2026.05.14.725288)
Supplement: Supplement 1 — Supplementary Table S1. GC×GC-MS metabolite quantification in MCF-7 and T47D breast cancer cells. Pairwise Welch's t-test results with Benjamini–Hochberg FDR correction for each of three pairwise comparisons (low glucose vs. high glucose; BHB-supplemented vs. high glucose; BHB-supplemented vs. low glucose) in each cell line. Reports log2 fold change, fold change, raw p-value, adjusted p-value, within-group coefficients of variation, and direction of change for each detected metabolite. [file media-1.pdf]

Supplementary Table S1. GC×GC-MS metabolite quantification in MCF-7 and T47D breast cancer cells

*Welch's t-tests with Benjamini–Hochberg FDR correction. MCF-7 analysis (n=8 per group); T47D analysis (n=14–15 per group). Significance coding: **red** = FDR p<0.05 with |FC|>1.5; **pink** = nominal p<0.05; **yellow** = trend (p<0.15 with |FC|>1.5). FC = fold change; CV = coefficient of variation.*

A. MCF-7 cells (n=8 per group)

A1. Low Glucose vs High Glucose (B vs A) — 46 metabolites tested

Metabolite	Log_2FC	FC	p-value	Adj. p-value	CV Ctrl (%)	CV Trt (%)	Direction
Isopropyl alcohol	0.85	1.80	0.1634	0.9434	91.1	68.7	↑
p-Xylene	0.32	1.25	0.1901	0.9434	24.8	34.7	↑
Pentane, 3-methyl-	0.53	1.44	0.2006	0.9434	3.2	28.4	↑
n-Hexane	0.13	1.10	0.2034	0.9434	1.0	8.1	↑
Cyclopentane, methyl-	0.24	1.18	0.2132	0.9434	4.9	15.2	↑
Pentane, 2-methyl-	-0.10	0.93	0.2541	0.9434	7.3	4.9	↓
Lactic Acid	-1.44	0.37	0.2574	0.9434	141.7	101.1	↓
Pentane, 2-methyl-	0.21	1.16	0.2624	0.9434	16.4	29.0	↑
2-(Dimethylamino)ethanol	0.79	1.73	0.3100	0.9434	47.1	53.5	↑
1,4-Butanediol	-0.72	0.61	0.3195	0.9434	69.6	90.1	↓
Pyrazine	0.32	1.25	0.3463	0.9434	55.4	34.6	↑
L-Valine	-0.92	0.53	0.3771	0.9434	72.2	46.5	↓
Silanol, trimethyl-	0.90	1.86	0.3838	0.9434	33.4	72.2	↑
Isopropyl alcohol	-0.44	0.74	0.4005	0.9434	28.4	52.9	↓
Acetic acid	-1.45	0.37	0.4106	0.9434	122.2	235.5	↓
1,3-Propanediol	0.99	1.99	0.4116	0.9434	33.8	83.5	↑
Butanoic acid, 4-hydroxy-	1.14	2.21	0.4302	0.9434	53.5	96.1	↑
Pyrazine	0.18	1.13	0.4565	0.9434	8.1	21.5	↑
Lactic Acid	0.40	1.32	0.4839	0.9434	59.9	29.4	↑
Ethylbenzene	0.32	1.24	0.5066	0.9434	36.1	36.3	↑
Ethanolamine	-0.64	0.64	0.5311	0.9434	97.5	79.4	↓
2,2,2-Trifluoroethane-1,1-diol, 2TMS	-0.96	0.51	0.5421	0.9434	166.2	234.3	↓
Glycine	-0.64	0.64	0.5925	0.9434	89.3	117.4	↓
2-(Dimethylamino)ethanol	-0.49	0.71	0.5993	0.9434	134.4	89.6	↓
Methoxyamine	-0.23	0.86	0.6189	0.9434	36.3	33.8	↓
Butanoic acid, 4-hydroxy-	0.73	1.66	0.6204	0.9434	204.7	178.4	↑
Cyclopentane, methyl-	0.09	1.07	0.6446	0.9434	29.9	23.8	↑
Ethylbenzene	-0.50	0.71	0.6802	0.9434	158.3	132.1	↓
Pentane, 3-methyl-	0.07	1.05	0.6903	0.9434	21.0	26.3	↑
Methylamine, N,N-dimethyl-	-0.24	0.85	0.6903	0.9434	57.7	1.0	↓
Ethylene glycol	0.09	1.06	0.7072	0.9434	6.1	22.6	↑
Pyridine	-0.23	0.85	0.7602	0.9434	93.4	72.0	↓
Methylamine, N,N-dimethyl-	-0.32	0.80	0.7646	0.9434	71.3	108.0	↓
Silanol, trimethyl-	0.26	1.20	0.7693	0.9434	131.8	100.4	↑
1,4-Butanediol	-0.08	0.95	0.7858	0.9434	22.6	22.7	↓
Methoxyamine	-0.36	0.78	0.7914	0.9434	137.1	186.6	↓
L-Alanine	-0.22	0.86	0.8008	0.9434	72.6	59.9	↓
Pyridine	-0.08	0.95	0.8254	0.9434	15.2	36.1	↓

Metabolite	Log_2FC	FC	p-value	Adj. p-value	CV Ctrl (%)	CV Trt (%)	Direction
Acetic acid	0.09	1.07	0.8495	0.9434	52.9	11.4	↑
Methylamine	-0.08	0.94	0.8556	0.9434	21.5	47.1	↓
Ethylene glycol	0.11	1.08	0.8558	0.9434	86.3	63.5	↑
2,2,2-Trifluoroethane-1,1-diol, 2TMS	-0.11	0.93	0.8613	0.9434	63.2	6.1	↓
1,3-Propanediol	-0.08	0.94	0.9393	0.9676	154.3	136.1	↓
Methylamine	-0.03	0.98	0.9434	0.9676	44.3	46.8	↓
p-Xylene	-0.04	0.97	0.9553	0.9676	11.4	72.6	↓
n-Hexane	0.00	1.00	0.9676	0.9676	7.4	10.0	↑

A2. BHB + Low Glucose vs High Glucose (C vs A) — 23 metabolites tested

Metabolite	Log_2FC	FC	p-value	Adj. p-value	CV Ctrl (%)	CV Trt (%)	Direction
Methylamine	0.60	1.52	0.0776	0.7061	44.3	23.8	↑
Silanol, trimethyl-	0.97	1.95	0.2087	0.7061	131.8	69.9	↑
Pyrazine	0.43	1.35	0.2192	0.7061	55.4	35.9	↑
p-Xylene	0.26	1.20	0.2209	0.7061	24.8	30.2	↑
Butanoic acid, 4-hydroxy-	1.65	3.15	0.2723	0.7061	204.7	139.3	↑
1,4-Butanediol	0.51	1.43	0.2881	0.7061	69.6	31.9	↑
1,3-Propanediol	0.94	1.92	0.2938	0.7061	154.3	87.7	↑
2-(Dimethylamino)ethanol	0.83	1.77	0.3147	0.7061	134.4	90.6	↑
Isopropyl alcohol	0.86	1.81	0.3303	0.7061	91.1	104.8	↑
Methylamine, N,N-dimethyl-	0.62	1.54	0.3353	0.7061	71.3	55.3	↑
Pentane, 2-methyl-	0.14	1.10	0.3377	0.7061	16.4	20.8	↑
Methoxyamine	0.73	1.66	0.4426	0.7443	137.1	79.2	↑
2,2,2-Trifluoroethane-1,1-diol, 2TMS	0.77	1.70	0.4451	0.7443	166.2	104.7	↑
Ethylene glycol	0.40	1.32	0.4530	0.7443	86.3	45.0	↑
Lactic Acid	0.60	1.52	0.5264	0.7675	141.7	115.9	↑
Ethanolamine	0.44	1.36	0.5919	0.7675	97.5	67.4	↑
Ethylbenzene	0.48	1.40	0.6347	0.7675	158.3	111.4	↑
n-Hexane	-0.02	0.98	0.6506	0.7675	7.4	6.9	↓
Pyridine	-0.38	0.77	0.6528	0.7675	93.4	92.0	↓
Glycine	0.38	1.30	0.6674	0.7675	89.3	69.5	↑
Cyclopentane, methyl-	-0.05	0.97	0.8478	0.9285	29.9	36.8	↓
Pentane, 3-methyl-	-0.02	0.99	0.9267	0.9596	21.0	26.7	↓
Acetic acid	-0.06	0.96	0.9596	0.9596	122.2	119.0	↓

A3. BHB + Low Glucose vs Low Glucose (C vs B) — 24 metabolites tested [KEY COMPARISON, COMPLETE]

Metabolite	Log_2FC	FC	p-value	Adj. p-value	CV Ctrl (%)	CV Trt (%)	Direction
1,4-Butanediol	1.23	2.35	0.0155	0.3726	90.1	31.9	↑
Methylamine	0.63	1.55	0.0568	0.5723	46.8	23.8	↑
Lactic Acid	2.05	4.13	0.1098	0.5723	101.1	115.9	↑
2-(Dimethylamino)ethanol	1.31	2.48	0.1167	0.5723	89.6	90.6	↑
2,2,2-Trifluoroethane-1,1-diol, 2TMS	1.73	3.32	0.1511	0.5723	234.3	104.7	↑
Ethanolamine	1.09	2.12	0.1655	0.5723	79.4	67.4	↑

Metabolite	Log_2FC	FC	p-value	Adj. p-value	CV Ctrl (%)	CV Trt (%)	Direction
1,3-Propanediol	1.02	2.03	0.2384	0.5723	136.1	87.7	↑
Glycine	1.02	2.03	0.2452	0.5723	117.4	69.5	↑
Methoxyamine	1.08	2.12	0.2634	0.5723	186.6	79.2	↑
Silanol, trimethyl-	0.71	1.63	0.2795	0.5723	100.4	69.9	↑
Methylamine, N,N-dimethyl-	0.94	1.92	0.3023	0.5723	108.0	55.3	↑
Ethylbenzene	0.98	1.97	0.3069	0.5723	132.1	111.4	↑
Acetic acid	1.39	2.62	0.3100	0.5723	235.5	119.0	↑
Butanoic acid, 4-hydroxy-	0.92	1.89	0.4657	0.7983	178.4	139.3	↑
Ethylene glycol	0.29	1.22	0.5134	0.8011	63.5	45.0	↑
Cyclopentane, methyl-	-0.14	0.91	0.5390	0.8011	23.8	36.8	↓
L-Valine	0.36	1.28	0.5723	0.8011	43.2	70.5	↑
Pentane, 3-methyl-	-0.09	0.94	0.6576	0.8011	26.3	26.7	↓
n-Hexane	-0.03	0.98	0.6773	0.8011	10.0	6.9	↓
Pentane, 2-methyl-	-0.07	0.95	0.6939	0.8011	29.0	20.8	↓
Pyrazine	0.11	1.08	0.7010	0.8011	34.6	35.9	↑
Pyridine	-0.14	0.91	0.8152	0.8569	72.0	92.0	↓
p-Xylene	-0.05	0.96	0.8212	0.8569	34.7	30.2	↓
Isopropyl alcohol	0.01	1.01	0.9906	0.9906	68.7	104.8	↑

B. T47D cells (all sets, n=14–15 per group)

B1. Low Glucose vs High Glucose (B vs A) — 24 metabolites tested

Metabolite	Log_2FC	FC	p-value	Adj. p-value	CV Ctrl (%)	CV Trt (%)	Direction
Nonadecane	0.19	1.14	0.0105	0.2519	13.3	12.5	↑
Propanedioic acid	0.42	1.34	0.0342	0.2749	24.9	38.8	↑
Heptacosane	0.15	1.11	0.0344	0.2749	13.1	12.1	↑
Nonadecane	0.12	1.09	0.0894	0.4755	13.1	12.4	↑
Pentane, 2-methyl-	0.07	1.05	0.1058	0.4755	4.9	8.9	↑
2,4,4,6-Tetramethyl-[1,3,2]dioxaborinane	-2.02	0.25	0.1189	0.4755	165.8	150.8	↓
Toluene	-3.36	0.10	0.1607	0.5044	227.0	66.4	↓
2,4-Di-tert-butylphenol	0.10	1.07	0.1730	0.5044	15.0	12.5	↑
Hydroxylamine, O-methyl-	0.10	1.07	0.1984	0.5044	11.6	15.0	↑
Cyclopentane, methyl-	-3.02	0.12	0.2102	0.5044	248.6	125.5	↓
1,1-Dimethylethanol	-0.44	0.74	0.2749	0.5997	81.4	44.9	↓
n-Hexane	0.19	1.14	0.3353	0.6706	37.7	33.1	↑
Acetic acid	-0.11	0.92	0.4187	0.6910	29.6	19.7	↓
β-D-(-)-Ribopyranose	0.06	1.04	0.4346	0.6910	13.5	14.1	↑
Glycine	-0.50	0.71	0.4365	0.6910	126.6	79.7	↓
Methylphosphonic acid	0.26	1.20	0.4728	0.6910	59.7	70.5	↑
α-Hydroxyisobutyric acid	0.08	1.06	0.4895	0.6910	19.8	21.4	↑
Butanoic acid, 2-methylbutyl ester	0.07	1.05	0.5328	0.7104	20.3	21.6	↑
N,N-Dimethyltrifluoroacetamide	0.08	1.06	0.5845	0.7384	23.7	28.5	↑
Hexane, 2-chloro-	-1.00	0.50	0.6364	0.7637	353.2	335.0	↓
Glycerol	0.05	1.03	0.6724	0.7685	21.2	21.2	↑
Lactic Acid	-0.12	0.92	0.7527	0.8211	84.5	53.3	↓
Acetaldehyde	-0.02	0.99	0.7928	0.8272	14.7	12.1	↓
Glycine	0.01	1.01	0.9000	0.9000	19.3	15.6	↑

B2. BHB + Low Glucose vs High Glucose (C vs A) — 24 metabolites tested

Metabolite	Log_2FC	FC	p-value	Adj. p-value	CV Ctrl (%)	CV Trt (%)	Direction
α-Hydroxyisobutyric acid	0.27	1.20	0.0606	0.4219	19.8	27.3	↑
β-D-(-)-Ribopyranose	0.16	1.12	0.1020	0.4219	13.5	19.3	↑
Butanoic acid, 2-methylbutyl ester	0.22	1.17	0.1046	0.4219	20.3	26.5	↑
N,N-Dimethyltrifluoroacetamide	0.27	1.20	0.1099	0.4219	23.7	32.1	↑
Propanedioic acid	0.30	1.23	0.1216	0.4219	24.9	38.3	↑
Methylphosphonic acid	0.77	1.70	0.1646	0.4219	59.7	100.2	↑
n-Hexane	0.26	1.20	0.1831	0.4219	37.7	32.7	↑
Heptacosane	0.10	1.07	0.2073	0.4219	13.1	15.5	↑
Nonadecane	0.10	1.07	0.2082	0.4219	13.3	15.5	↑
Nonadecane	0.10	1.07	0.2093	0.4219	13.1	15.5	↑
1,1-Dimethylethanol	-0.50	0.71	0.2153	0.4219	81.4	31.6	↓
Cyclopentane, methyl-	-2.91	0.13	0.2155	0.4219	248.6	133.4	↓
Toluene	-2.20	0.22	0.2285	0.4219	227.0	235.3	↓
2,4-Di-tert-butylphenol	0.10	1.07	0.2527	0.4332	15.0	15.9	↑

Metabolite	Log_2FC	FC	p-value	Adj. p-value	CV Ctrl (%)	CV Trt (%)	Direction
Hexane, 2-chloro-	2.59	6.02	0.2850	0.4560	353.2	274.6	↑
Hydroxylamine, O-methyl-	0.05	1.04	0.4269	0.6233	11.6	12.9	↑
Glycerol	0.10	1.07	0.4415	0.6233	21.2	25.0	↑
2,4,4,6-Tetramethyl-[1,3,2]dioxaborinane	-0.78	0.58	0.5171	0.6895	165.8	295.7	↓
Lactic Acid	-0.22	0.86	0.5898	0.7124	84.5	56.6	↓
Acetic acid	0.08	1.06	0.5937	0.7124	29.6	28.1	↑
Pentane, 2-methyl-	0.01	1.01	0.6751	0.7716	4.9	7.4	↑
Glycine	0.11	1.08	0.8768	0.9433	126.6	133.5	↑
Acetaldehyde	0.01	1.01	0.9274	0.9433	14.7	14.6	↑
Glycine	0.01	1.01	0.9433	0.9433	19.3	18.4	↑

B3. BHB + Low Glucose vs Low Glucose (C vs B) — 24 metabolites tested [KEY COMPARISON]

Metabolite	Log_2FC	FC	p-value	Adj. p-value	CV Ctrl (%)	CV Trt (%)	Direction
Acetic acid	0.20	1.15	0.1546	0.8687	19.7	28.1	↑
α-Hydroxyisobutyric acid	0.19	1.14	0.1739	0.8687	21.4	27.3	↑
Hexane, 2-chloro-	3.59	12.03	0.2353	0.8687	335.0	274.6	↑
Nonadecane	-0.09	0.94	0.2553	0.8687	12.5	15.5	↓
Pentane, 2-methyl-	-0.05	0.97	0.2576	0.8687	8.9	7.4	↓
Butanoic acid, 2-methylbutyl ester	0.15	1.11	0.2608	0.8687	21.6	26.5	↑
N,N-Dimethyltrifluoroacetamide	0.19	1.14	0.2687	0.8687	28.5	32.1	↑
β-D-(-)-Ribopyranose	0.10	1.07	0.2906	0.8687	14.1	19.3	↑
Methylphosphonic acid	0.51	1.42	0.3289	0.8687	70.5	100.2	↑
Glycine	0.61	1.53	0.3777	0.8687	79.7	133.5	↑
Toluene	1.16	2.24	0.3981	0.8687	66.4	235.3	↑
2,4,4,6-Tetramethyl-[1,3,2]dioxaborinane	1.23	2.35	0.4886	0.9239	150.8	295.7	↑
Heptacosane	-0.05	0.97	0.5252	0.9239	12.1	15.5	↓
Propanedioic acid	-0.12	0.92	0.5560	0.9239	38.8	38.3	↓
Hydroxylamine, O-methyl-	-0.04	0.97	0.5898	0.9239	15.0	12.9	↓
n-Hexane	0.08	1.05	0.6701	0.9239	33.1	32.7	↑
Glycerol	0.05	1.04	0.6872	0.9239	21.2	25.0	↑
Acetaldehyde	0.03	1.02	0.7162	0.9239	12.1	14.6	↑
Lactic Acid	-0.10	0.93	0.7430	0.9239	53.3	56.6	↓
1,1-Dimethylethanol	-0.06	0.96	0.7887	0.9239	44.9	31.6	↓
Nonadecane	-0.02	0.99	0.8084	0.9239	12.4	15.5	↓
Cyclopentane, methyl-	0.12	1.08	0.8703	0.9494	125.5	133.4	↑
2,4-Di-tert-butylphenol	-0.00	1.00	0.9564	0.9604	12.5	15.9	↓
Glycine	-0.00	1.00	0.9604	0.9604	15.6	18.4	↓
