# Supplementary material for: β-Hydroxybutyrate elicits divergent metabolic responses between MCF-7 and T47D ER+ breast cancer cells under glucose restriction
Source: bioRxiv. 2026 May 18:2026.05.14.725288. Preprint. [Version 1] doi: 10.64898/2026.05.14.725288 (PMC13228522; doi:10.64898/2026.05.14.725288)
Supplement: Supplement 2 — Supplementary Table S2. Complete pairwise correlation matrix for SHMT1, SHMT2, and BCAT1 versus OXCT1, ACAT1, and BDH1 in the TCGA Breast Invasive Carcinoma cohort (n = 1,084 samples). Pearson and Spearman coefficients with corresponding p-values are reported for all nine gene pairs; the four pairs meeting both Bonferroni-corrected significance (α = 0.0056) and a minimum correlation magnitude (r > 0.10) are highlighted and shown individually in Figure 4 of the main manuscript. [file media-2.pdf]

Supplementary Table S2. TCGA Breast Invasive Carcinoma co-expression analysis (n=1,084 samples)

Pairwise correlations between one-carbon/BCAA metabolism genes (SHMT1, SHMT2, BCAT1) and ketone body catabolic genes (OXCT1, ACAT1, BDH1). Source: cBioPortal, TCGA PanCancer Atlas, mRNA expression (RSEM, batch normalized, log_2). Bonferroni correction for 9 simultaneous tests: $\alpha = 0.05/9 = 0.0056$. The four pairs meeting both Bonferroni-corrected significance and a minimum correlation magnitude ($|r| > 0.10$) are highlighted.

Gene 1	Gene 2	Pearson r	Pearson p	Spearman ρ	Spearman p	Meets Bonferroni + |r| > 0.10
SHMT1	BDH1	+0.18	2.88e-9	+0.20	2.16e-11	**Yes**
SHMT1	OXCT1	+0.03	0.4000	−0.01	0.7000	No
SHMT1	ACAT1	+0.05	0.1330	+0.07	0.0312	No
SHMT2	BDH1	+0.06	0.0606	+0.07	0.0218	No
SHMT2	OXCT1	+0.17	3.46e-8	+0.13	1.17e-5	**Yes**
SHMT2	ACAT1	−0.03	0.4000	−0.03	0.3970	No
BCAT1	BDH1	−0.36	3.69e-35	−0.40	1.43e-43	**Yes**
BCAT1	OXCT1	+0.16	1.10e-7	+0.12	5.82e-5	**Yes**
BCAT1	ACAT1	+0.03	0.2870	+0.05	0.0712	No

The strongest association is BCAT1–BDH1 (Spearman $\rho = -0.40$, $R^2 = 0.13$); the remaining three significant pairs are weak in magnitude ($R^2 \approx 0.03$) and are interpreted as exploratory transcriptional associations rather than evidence of direct mechanistic coupling.
