# Supplementary figures and images for: β-Hydroxybutyrate elicits divergent metabolic responses between MCF-7 and T47D ER+ breast cancer cells under glucose restriction
Source: bioRxiv. 2026 May 18:2026.05.14.725288. Preprint. [Version 1] doi: 10.64898/2026.05.14.725288 (PMC13228522; doi:10.64898/2026.05.14.725288)

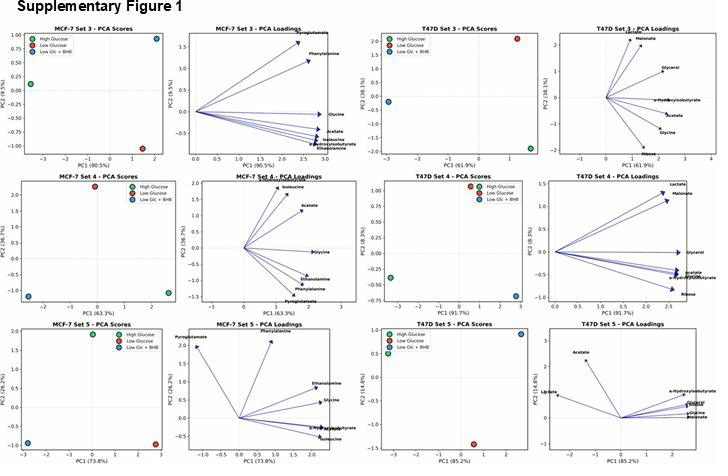

Supplement: Supplement 3 — Supplementary Figure S1. Individual experimental set PCA analysis showing PCA scores (left panels per set) and loadings (right panels per set) for MCF-7 (sets 3, 4, 5) and T47D (sets 3, 4, 5) breast cancer cells under high glucose, low glucose, and low glucose with BHB conditions. Treatment-dependent directional separation is consistent across individual experimental sets despite between-batch technical variability. [file media-3.jpg]
